# Abstract of a Government Report on the Teeth of Public School Children in New South Wales

**Published:** 1910-02-15

**Authors:** William Rushton


### ABSTRACT OF A GOVERNMENT REPORT ON THE TEETH OF PUBLIC SCHOOL CHILDREN IN NEW SOUTH WALES.

BY WILLIAM RUSHTON, L.D.S., ENG.

There has been recently issued by the Department of Public Instruction, New South Wales, a Blue Book, which is a Report on the Physical Condition of Children attending the Public Schools in New South Wales. The Report goes very fully into the statistics obtained as a result of the introduction of a scheme of Medical Inspection of the Public School Children, with special reference to height, weight and vision, and many interesting anthropometric tables and diagrams are given. We shall not enlarge upon these further than to say that the country-bred children are finer specimens than the town-bred ones; that the children of our professional classes have very similar measurements to those of the New South Wales well-to-do classes, the girls of the latter having a slight advantage; and that speaking generally, our children of the labouring class fall far below the New South Wales standard of the similar class, at all ages. The figures for the similar class in Germany are not given, though, judging from the limited number of well-to-do German children measured, it would appear that they are a little heavier, although very much shorter, than the average New South Wales child.

With respect to vision, the results are very fair. Those children coming from the seaside and country schools have the best sight; those with weakest sight come from schools in the poorer city areas. Of the total number of children examined, 93 per cent. of the boys and 88 per cent. of the girls may be said to possess good sight.

### TEETH.

The inspection of the teeth of Sydney school children was undertaken gratuitously by the Dental Association of New South Wales and commenced in 1904. Every facility was placed at their disposal by the Education Department and charts were supplied by the Government Printing Office. Two charts were employed for each child, one was kept as a record by the Department, the other was forwarded to the parent of the child, with instructions on the care of the teeth. The general results are as follows:

| | Boys. | Girls. | Total. |
|---|---|---|---|
| Number of permanent teeth examined | 49,820 | 26,625 | 76,445 |
| " temporary " " | 11,766 | 6,470 | 18,236 |
| " permanent " carious | 8,259 | 5,283 | 13,542 |
| " temporary " " | 3,421 | 2,271 | 5,692 |

This statement indicates that, in the case of boys, 16 per cent. of the permanent teeth and 29 per cent. of the first set were found to be carious. In the case of girls, 19 per cent, of the permanent teeth and 35 per cent. of the first set were found to be carious.

The teeth of girls are thus seen to be more defective than those of boys, both as regards the first and second sets. Of all the permanent teeth examined (both sexes), 17 per cent.

were found to be carious. Of the temporary teeth, 31 per cent. were carious. It is important to notice that defects are very much more common among teeth of the first set than among the permanent teeth.

Then follow some elaborate tables of the condition of the teeth and mouths of children at each age from five to nineteen years, from which we gather that of the 2,631 boys examined, 802, or 30 per cent. had clean mouths. In other words, they used the tooth-brush with some degree of regularity. 1,485 boys, or 57 per cent of the total number examined, had fairly clean mouths, while 344 boys, or 13 per cent. had dirty mouths. Of the girls, 32 per cent, had clean mouths, 56 per cent, had fairly clean mouths, and 12 per cent. had dirty mouths.

Cases of extraction of teeth and teeth-filling were most frequently found amongst pupils of both sexes between the ages of 13 and 15 years.

The average number of defective teeth per boy was found to be 4.4, and per girl 5.2. Of the teeth of all children examined, 20.3 per cent. were found to be defective. Of the boys' teeth (both sets), 18 per cent. were found to be defective. Of the girls' teeth (both sets), 22 per cent. were found to be defective.

In some cases teeth of the first set remained amongst boys and girls of 12, 13, and even 16 years.

Two periods are noted at which the teeth are specially defective. The first, from six to seven, is common to both sexes and is "apparently associated with the second dentition and the shedding of the first teeth." The second period, 14 to 16 in boys, and 12 to 15 in girls, "is apparently associated with the pubertal changes." These suggested causes are open to criticism, especially the latter; in fact, the next paragraph hints as much, as it says, "these are the very years" (12 and 14 in boys and girls respectively) "when the neglect of the tooth brush is most apparent."

The report then goes on to quote various opinions and statistics. Dr. Sims Lever, of the N. S. W. Dental Association, says that "Analysis shows that 94 per cent. of the Sydney children examined have decayed teeth." "Dr. Pedley" (probably Mr. Denison Pedley) "reports that about 75 per cent, of the children of the poorer classes in England have decayed teeth."

"Another authority gives 86 per cent. in an examination of 15,000 children, and 97 per cent. in a further examination of 1,200 children. In Germany, out of 20,000 examined, from six to 15 years of age, 95 per cent, had dental caries; in Russia, the figures are 95 per cent. and in Italy, between 90 and 100 per cent. According to Dr. Ambler, over 90 per cent. of the children of the poorer classes in America have diseased teeth, and over 30 per cent. of all the teeth of children between five and 15 in the public schools of the United States are diseased."

"This examination, as far as it goes, shows that the percentage of children with diseased teeth (94 per cent.) is about the same in Sydney as in Germany, Russia, Italy, and the United States. The value of the comparison is, however, discounted by the fact that the child with one bad tooth is put in the same category as the child with several. Perhaps a more correct basis of comparison is furnished by the fact that in Sydney, 20.3 per cent. of the teeth examined were found to be defective, while in America the average is 30 per cent.

The report also draws attention to the paper contributed by Mr. J. G. Turner to the *British Medical Journal* of June 20th, 1908, in which he draws the conclusion that the etiology of the disease is dirt and the obvious prophylaxis is cleanliness. To some of our minds this statement, though excellent in its way, does not explain all the mystery of dental caries. As a good working formula for the public however, we would not quibble at it. In Mr. Turner's article, "two carious teeth per child are regarded as the average number of decayed teeth among the poor children of London. Our Sydney figures are much higher, namely, four carious teeth per boy and five per girl." An Edinburgh report "shows that the proportion of children with dirty mouths in Edinburgh is 25 per cent., or just double that of Sydney children, namely, 12½ per cent." The proportion of decayed teeth of the first and second set is, however, considerably lower in Edinburgh than in Sydney, which seems rather a heavy blow to the "cleanliness" theory.

### SUMMARY.

The following summary is appended to the report, and gives in a few lines several years of work:

1. Records of inspection of the teeth of 4,076 children in the metropolitan district were made by the Dental Association of New South Wales.

2. The teeth of girls are more defective than those of boys as regards both the first and second sets. Defects are much more common among teeth of the first set than among permanent teeth.

3. Of all permanent teeth examined, 17 per cent. were found to be carious.

4. Of all temporary teeth examined, 31 per cent. were found to be carious.

5. Of the boys examined, 30 per cent. had clean mouths (that is, used the tooth-brush regularly), 57 per cent. fairly clean mouths, and 13 per cent. dirty mouths.

6. Of the girls examined, 32 per cent. had clean mouths, 56 per cent. fairly clean mouths, and 12 per cent. dirty mouths.

7. The average number of defective teeth was 4.4 per boy and 5.2 per girl.

8. The two periods in child-life when the teeth are most defective occur—(*a*) about ages six and seven; and (*b*) from 14 to 16 (boys), and from 12 to 15 (girls). The first period is related to the shedding of the first teeth, the second to the pubertal changes.

9. The period of rapid decay are the periods when the neglect of the tooth-brush is most apparent.

10. The percentage of children with defective teeth in Sydney (94) is about the same as in Germany, Russia, Italy and America.

11. A comparison of the teeth of Sydney children with those of London, Edinburgh, and Aberdeen, shows that the former are the cleaner. The number of decayed teeth is, however, less per child in England and Scotland than in New South Wales.

The profession must feel grateful to the Dental Association of New South Wales and to their Government for the work and willing help which have produced this report. We cannot have too many correct figures. Given a sufficiency of these, correct deductions must follow as the night the day, and they, in their turn, are sure to be followed by the amelioration of the evil.

Since writing the above the following reports from Preston and Manchester have come to hand, and will prove interesting:

PRESTON.

In his first annual report Dr. C. Murray, Medical Officer under the Preston Education Committee, says that out of 1,404 children visited he found defects of one kind or another in 1,125. Dental diseases were found among 72 per cent., a figure which he fears will be much higher when a larger number of children are dealt with. Anaemia was the next most prevalent defect, and he believed it to be largely due to carious teeth, but improper and insufficient food and fatigue among half-timers were also contributory causes. Defective vision was found in about 14 per cent. of the children. Dr. Murray says it would be an admirable thing to have the hair of every child attending a public elementary school cut short, or at any rate whenever vermin was found.

The height of the children was practically one inch below the average standard of Great Britain, but he did not attach very great importance to that. A matter which was, however, most important, and to which he desired to draw attention of the public, was that of the weight of the children. They found that in the case of children from three to four years they were, as compared with the standard of Great Britain, 1 lb. to 1½ lb. lighter. That was not very much, but as they went up the years they found that between the ages of 13 and 14 there was a deficiency of 9 lbs. as compared with the standard throughout the kingdom. To his mind, that was very important and serious position of affairs, and one which the parents of Preston may alter within a reasonable time if they would only apply the remedy.

MANCHESTER.

The statement in last year's report that, so far as the analysis of the investigation had been carried out, the Medical Officer was unable to state that the condition of the teeth is necessarily reflected in the physical condition of the child, has been borne out by the examination made this year. It was found that the smallest number of teeth among the girls was 12, and the great majority of them had 20 or more. Among the boys the smallest number was 11, and the great majority has 20 or more. Of 281 boys examined, 13.9 per cent· had dirty teeth; of those with sound teeth 7.7 per cent. had teeth in a dirty condition. Of 508 girls examined 17.5 per cent. had dirty teeth, while those who had sound teeth, 48 per cent. had teeth in a dirty condition. The value of the figures with regard to dirty teeth is greatly reduced by the fact that after the first day's examination, many of the children came with their teeth brushed, often probably for the first time, and when examined were not in their ordinary condition.

The Medical Officer adds that although it would appear from the evidence that the condition of the child's teeth is not of great importance, this would be a dangerous interpretation of the statistics. Were the condition of those having a large number of defective teeth to be investigated when the children had reached 17 years of age, he is of opinion that allowing for the many new factors introduced into the child's life, and which would militate against the normal condition of health, reliable statistics would show a definite deterioration directly due to defective teeth. The question of the care of children's teeth is a very large one. The percentage of those having defective teeth is exceedingly high, and many of them have a large number of teeth in an advanced state of decay. It was obvious that the state of the mouth and teeth in a fair number of the children was such that every facility was given for the development of disease; yet in a very large proportion of them no such result obtained at the time of examination. The result of individual examination will be that the parents' attention will at least be drawn to cases similar to those which were circularised after the above investigation, an attempt will be made to persuade them to have the defects remedied.

Mr. Charles E. Hecht, Secretary of the National Food Reform Association, writes from 178, St. Stephen's House, Victoria Embankment, Westminster Bridge, London, S. W.:—"In view of Dr. Ritchie's depressing report upon the physical condition of Manchester children, and more particularly his remarks on the bad state of their teeth, it may not be without interest to point out that Dr. Harry Campbell, an acknowledged authority in the medical world on dietic matters, speaking at a recent meeting in London, denounced the condition of the teeth of the poor of the Metropolis as a disgrace to the country. He largely attributed it to insufficient mastication and to the excessive consumption of starch, especially in patent foods, holding the former practice, mainly responsible among other evils, of adenoids among children (cp. Dr. Ralph Crowley's valuable report for 1908 to the Bradford Education Committee, page 57), and for the increase in dental caries or disease of the sockets in the teeth of adults. Dr. Albert Wilson, while endorsing Dr. Campbell's remarks, also largely attributed defective teeth to the lax administration of the law relating to milk. He found, on a recent visit to a Dutch reformatory, that practically all the boys had good teeth, the reason being that the Dutch poor get good milk as well as plenty of butter, cream, and cheese. Indeed, in Holland, bad teeth were the exception among children; in our slums they were the rule. Facts like these are calculated to increase the disappointment which so many social reformers are feeling at the failure of the Government to pass Mr. John Burns's Milk Bill this session. Having regard to the profound influence which attention to diet exerts on the condition of the teeth, some of your readers may be glad to know that the National Food Reform Association have prepared a penny booklet, entitled "Economical Dishes for Workers," which contains, in addition to recipes, hints on the subject of mastication, the feeding of children, and other matters."

After reading these various reports one cannot help arriving at the conclusion—borne out by clinical observation—that it is not the dirtiest mouths which necessarily contain the most carious teeth, and conversely that many industrious users of the tooth-brush have many carious teeth. Milk, too, which is so highly and deservedly lauded has been suggested by Goadby as a cause of pyorrhea alveolaris. We have much to learn!—*Dental Record.*

